# Correction to “Disordered p53‐MALAT1 Pathway is Associated With Recurrent Miscarriage”

**DOI:** 10.1002/kjm2.70150

**Published:** 2025-12-05

Y. Wang, H. Z. Liu, Y. Liu, H. J. Wang, W. W. Pang, and J. J. Zhang, “Disordered p53‐MALAT1 Pathway is Associated With Recurrent Miscarriage,” *Kaohsiung Journal of Medical Sciences* 35 (2019): 87–94, https://doi.org/10.1002/kjm2.12013.

In our published article “Disordered p53‐MALAT1 pathway is associated with recurrent miscarriage” (PMID: 30848022, 2019), we identified errors in Figure 4 that require correction. Specifically, panels D and F contained incorrect cell line labels, and panels D and E had partial image overlaps between the control and pcDNA3.1‐p53+MALAT1 treatment groups. These errors occurred inadvertently during figure assembly and image management and are confined to this single figure; all images are original, unaltered microscopic captures. Importantly, these issues do not affect the scientific conclusions of the study. To ensure clarity and accuracy for readers, we have corrected the figure by providing the appropriate cell line labels and non‐overlapping images, and the figure legend has been updated accordingly. We sincerely apologize for these oversights.

**FIGURE 4** MALAT1 overexpression partially restored p53 function in HTR‐8/SVneo cells and villus trophoblasts. HTR‐8/SVneo cells were transfected with p53 expression vector with or without MALAT1 expression plasmid for 48 hours. The cells were subjected to MTT assay (A), flowcytometry analysis after PI and annexin V‐fluorescein staining (B), migration assay (C and E), and invasion assay (D and F). The results were analyzed by Student *t* test. *p* < 0.05 was considered statistically significant. *p < 0.05, **p < 0.01. PI, prepidium iodide.
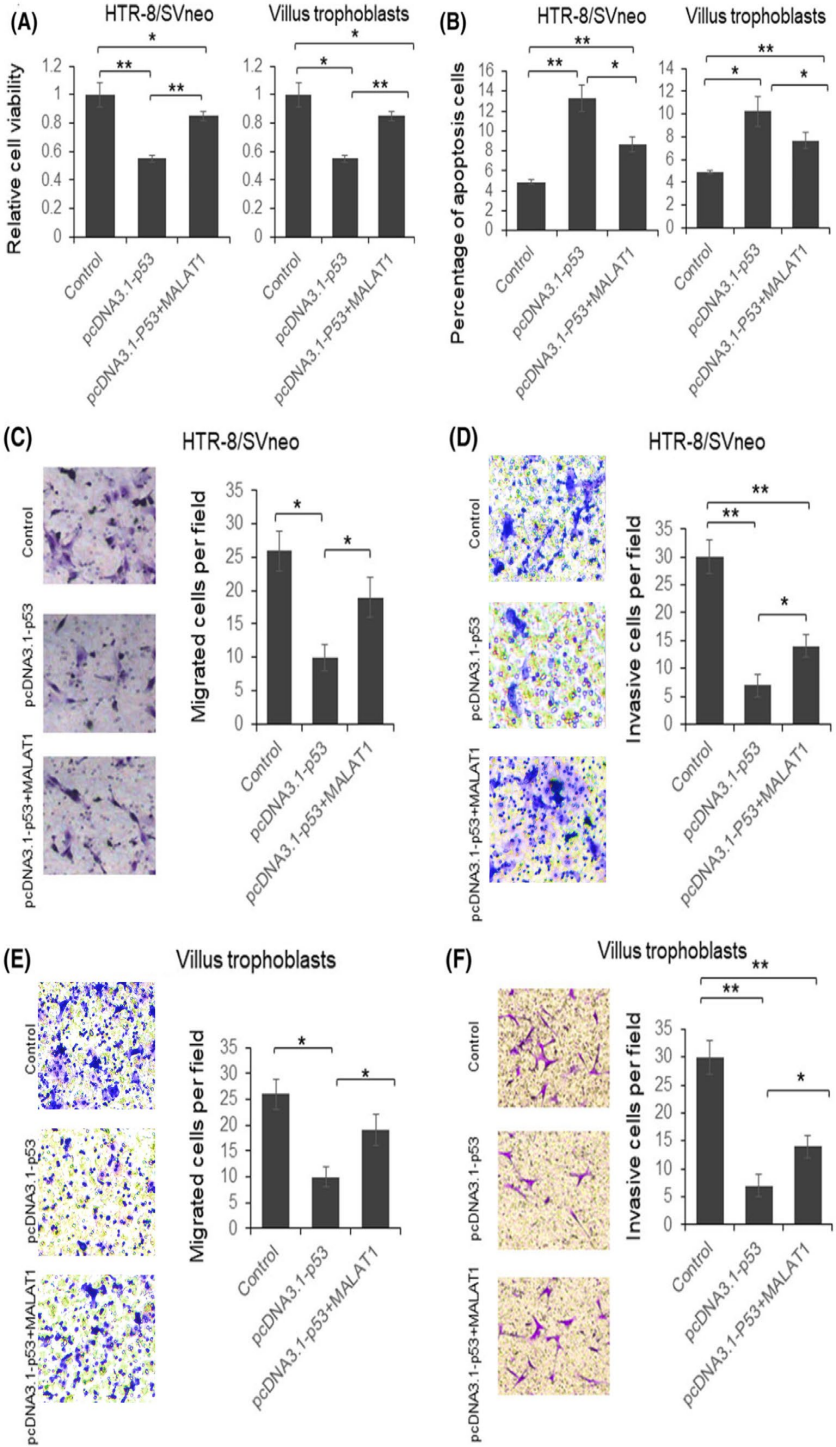


The authors confirmed that all results and conclusions of this article remain unchanged.

We apologize for this error.

